# Oral environment and cancer

**DOI:** 10.1186/s41021-016-0042-z

**Published:** 2016-08-01

**Authors:** Yasusei Kudo, Hidesuke Tada, Natsumi Fujiwara, Yoshiko Tada, Takaaki Tsunematsu, Yoichiro Miyake, Naozumi Ishimaru

**Affiliations:** 1 Department of Oral Molecular Pathology, Institute of Biomedical Sciences, Tokushima University Graduate School, Tokushima, Japan; 2 Department of Oral Healthcare Promotion, Institute of Biomedical Sciences, Tokushima University Graduate School, Tokushima, Japan; 3 Tada Dental Clinic, Kakogawa, Japan; 4 Department of Oral Microbiology, Institute of Biomedical Sciences, Tokushima University Graduate School, Tokushima, Japan

**Keywords:** Cancer, Oral environment, Bacteria, *Candida*, Human papilloma virus, Epstein-Barr virus

## Abstract

Cancer is now the leading cause of death in Japan. A rapid increase in cancer mortality is expected as Japan is facing a super-aged society. Many causes of cancer are known to be closely linked to life style factors, such as smoking, drinking, and diet. The oral environment is known to be involved in the pathogenesis and development of various diseases such as bronchitis, pneumonia, diabetes, heart disease, and dementia. Because the oral cavity acts as the bodily entrance for air and food, it is constantly exposed to foreign substances, including bacteria and viruses. A large number of bacteria are endemic to the oral cavity, and indigenous oral flora act to prevent the settlement of foreign bacteria. The oral environment is influenced by local factors, including dental plaque, tartar, teeth alignment, occlusion, an incompatible prosthesis, and bad lifestyle habits, and systemic factors, including smoking, consumption of alcohol, irregular lifestyle and eating habits, obesity, stress, hormones, and heredity. It has recently been revealed that the oral environment is associated with cancer. In particular, commensal bacteria in the oral cavity are involved in the development of cancer. Moreover, *Candida*, human papilloma virus and Epstein-Barr virus as well as commensal bacteria have been reported to be associated with the pathogenesis of cancer. In this review, we introduce recent findings of the correlation between the oral environment and cancer.

## Background

During 2012, approximately 14.1 million new cases of human cancer were reported globally [1]. In Japan, the estimated number of cancer deaths during 2014 was approximately 367,000 [2]. Regarding the age-specific causes of death, during 2013, cancer was the leading cause of death among 40–89 years in Japan [2]. Tobacco use accounts for approximately 22 % of cancer deaths [3], followed by obesity, a poor diet, lack of physical activity, and consumption of alcohol, which collectively account for 10 % of cancer deaths [3, 4]. Other factors include infections, exposure to ionizing radiation, and environmental pollutants [5]. Thus, various causes of cancer are known to be closely involved in lifestyle choices, such as smoking, drinking, and diet.

**Fig. 1:**
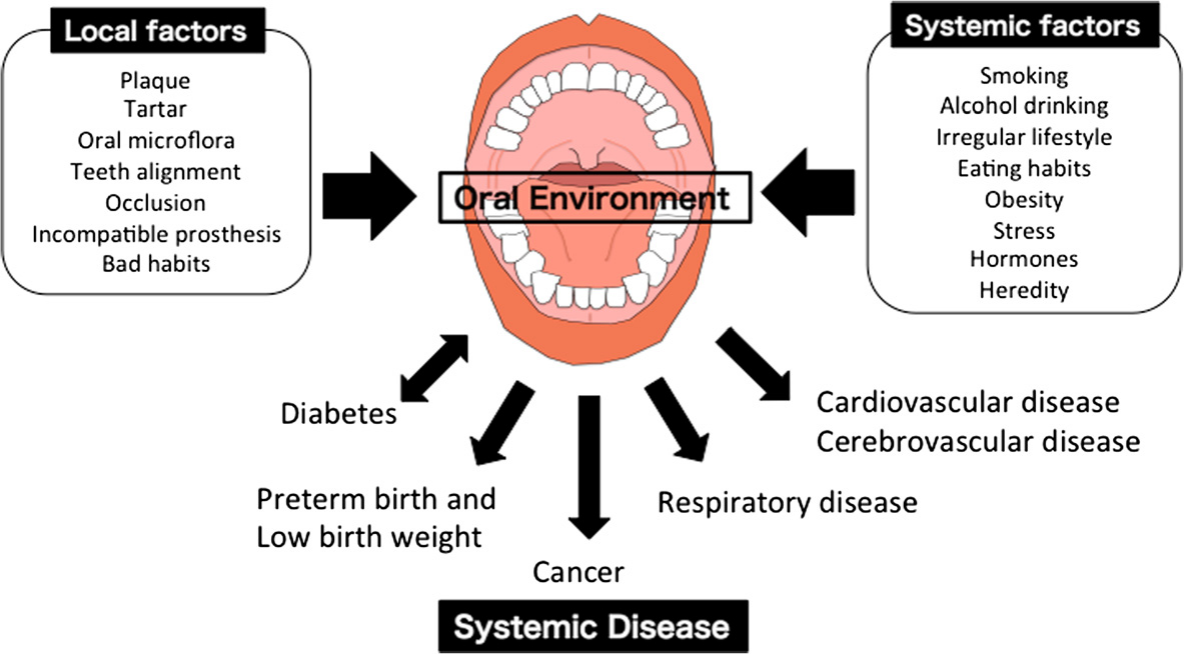
Influence of Oral Environment on Systemic Disease. The oral environment is influenced by local factors, including dental plaque, tartar, teeth alignment, occlusion, an incompatible prosthesis, and bad lifestyle habits, and systemic factors, including smoking, consumption of alcohol, an irregular lifestyle, eating habits, obesity, stress, hormones, and heredity factors. The oral environment is involved in systematic diseases, such as diabetes, preterm birth and low birth weight, cancer, respiratory disease, cardiovascular disease and cerebrovascular disease

The human body is inhabited by over 100 trillion microbial cells living in symbiosis with the host. Many bacteria are endemic to the oral cavity. More than 700 bacterial species inhabit the oral cavity, including at least 11 bacterial phyla and 70 genera. Individuals that practice oral hygiene have 1,000–100,000 bacteria living on each tooth surface, whereas those who do not regularly practice dental hygiene can have between 100 million and 1 billion bacteria on each tooth surface [6]. Although some bacteria of the oral cavity are harmful and can cause serious disease, many of the oral bacteria are in fact beneficial in preventing diseases. Thus, the oral cavity is inhabited by complex multispecies bacterial communities that usually exist in a balanced immunoinflammatory state with the host [7]. It is now established that many chronic inflammatory conditions are caused by an imbalance between host-microbiota interactions, resulting in a dysbiotic community, deregulated immune responses, and eventually disease outcomes. Oral commensal bacteria play a critical role in the development of oral diseases, including periodontal disease and tooth loss, and maintenance of a normal oral physiological environment [8, 9]. In addition, oral commensal bacteria are involved in the pathogenesis of cancer [10]. Thus, the oral environment including oral commensal bacteria is known to be involved in the pathogenesis and development of various diseases, such as bronchitis and pneumonia, diabetes, heart disease, and dementia. The oral environment is influenced by local factors, including dental plaque, tartar, teeth alignment, occlusion, an incompatible prosthesis, and bad lifestyle habits, and systemic factors, including smoking, consumption of alcohol, an irregular lifestyle, eating habits, obesity, stress, hormones, and heredity factors (Fig. 1). Majority of the causes of cancer are thought to be related to tobacco use and heavy alcohol consumption. In the oral environment, poor oral hygiene and viral and Candida infections can be risk factors for cancer.

## Oral bacteria and cancer

The link between oral infections and adverse systemic conditions has attracted a great deal of attention in the dental and medical fields. A major consequence of the systemic spread of oral commensals and pathogens to distant body sites has been the disruption of immune surveillance and homeostasis, resulting in the promotion or acceleration of pathogenic processes. Oral bacteria are involved in systemic infections and inflammation such as cardiovascular disease, adverse pregnancy outcomes, rheumatoid arthritis, aspiration pneumonia, inflammatory bowel disease, organ inflammations, and cancer (Fig. 1). Cumulative studies have revealed that oral bacteria are involved in the initiation or progression of certain cancers. Chronic or dysregulated inflammation contributes to tumor development, partly through the modulation of the tumor microenvironment [11]. Epidemiological studies have shown that the risk of cancer is increased in people with periodontal disease or tooth loss caused by oral bacteria [12]. Oral diseases, including periodontitis, have been related to the risk of oral and gastrointestinal cancers, such as oral, esophageal, gastric and pancreatic cancers [13, 14]. Although the underlying mechanism for the associations between oral health status and cancers are not completely understood, it has been reported that *Fusobacterium nucleatum* and *Porphyromonas gingivalis* are involved in cancer development.

**Fig. 2:**
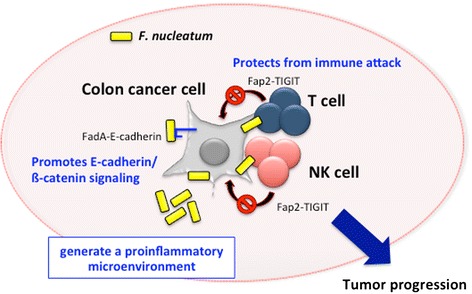
Involvement of *F. nucleatum* in colon cancer development. Figure shows the mechanisms of *F. nucleatum* in the involvement of colon cancer. *F. nucleatum* promotes E-cadherin/ß-catenin signaling via FadA adhesin and protects from immune attack by engaging its bacterial protein Fap2 with the inhibitory immunoreceptor TIGIT on NK and T cells. Moreover, *F. nucleatum* generate a proinflammatory microenvironment through the recruitment of tumor-infiltrating immune cells

*F. nucleatum*, an anaerobic Gram-negative oral commensal, is associated with periodontitis, adverse pregnancy outcomes, cardiovascular disease, rheumatoid arthritis, inflammatory bowel disease, and colon cancer [15]. This bacterial species is a key component of periodontal plaque because of its abundance and ability to coaggregate with other species in the oral cavity [16, 17]. Elevated *F. nucleatum* levels were significantly detected in people with colon cancers compared with that in people with normal colon tissue [18, 19]. The bacteria genera of *Fusobacterium, Porphyromonas, Peptostreptococcus*, and *Mogibacterium* were found to be enriched in the mucosa-adherent microbiota of people with colon cancer [20]. Moreover, the overabundance of *Fusobacterium* in people with colon cancer was positively associated with lymph node metastasis [18]. In cases of colon cancer, a positive correlation between mRNA levels for several local cytokines and *Fusobacterium* species has been observed [21]. Although the mechanism by which *F. nucleatum* might contribute to the pathogenesis of such a diverse clinical spectrum is unknown, recent studies have demonstrated the mechanisms of *F. nucleatum* in the involvement of colon cancer as follows: i) *F. nucleatum* induces oncogenic and inflammatory responses to stimulate growth of colon cancer cells through FadA adhesin via binding with E-cadherin and ensuing activation of ß-catenin signaling [22], ii) *F. nucleatum* generate a proinflammatory microenvironment for the progression of colon cancer through the recruitment of tumor-infiltrating immune cells [23], and iii) *F. nucleatum* interferes with the host immunity by engaging its bacterial protein Fap2 with the inhibitory immunoreceptor TIGIT on NK and T cells [24] (Fig. 2).

Accumulated evidence has shown strong correlations between a number of chronic periodontal bacteria containing *Prevotella*, *Porphyromonas* and *Streptococcus* spp. and orodigestive cancer, including oral squamous cell carcinoma and pancreatic cancer [25–31]. *P. gingivalis* have an abundance of virulence factors and can be present in periodontal pockets. *P. gingivalis* has a major disruptive effect on local immune responses in the periodontal area [7]. Moreover, it can survive within the oral epithelial tissues and can evade the host immune response [32–37]. *In vitro* studies have demonstrated that i) *P. gingivalis* strains can induce the expression of the B7-H1 and B7-DC receptors in oral cancer cells, which might facilitate immune evasion by oral cancers [38] and ii) *P. gingivalis* activates the ERK1/2-Ets1, p38/HSP27, and PAR2/NF-kB pathways to induce proMMP9 expression, after which the proenzyme is activated by gingipains to promote cellular invasion of oral cancer cells [39]. In primary cultures of gingival epithelial cells, *P. gingivalis* is strongly antiapoptotic and can suppress chemically induced apoptosis via activation of the Jak1/Akt/Stat3 signaling pathway [40, 41], suggesting that the antiapoptotic effect of *P. gingivalis* may be involved in carcinogenesis. Thus, both *P. gingivalis* and *F. nucleatum* are involved in cancer progression via activation of cell signaling and/or facilitating immune evasion. However, the possible link between oral bacterium and cancer development remains to be investigated in molecular detail.

## Viral and *Candida* infection and cancer

Tobacco and alcohol are considered as major risk factors for cancer; however, viral and candidal infection is increasingly being identified to play significant roles in cancer development [42]. Human papillomaviruses (HPVs) are small, double-stranded DNA viruses that induce hyperproliferative lesions in epithelial tissues [43, 44]. More than 100 types of HPV have been identified. Among them, high-risk HPV types, including HPV type 16 (HPV-16), HPV-18, HPV-31, HPV-33, and HPV-42, induce lesions in the genital tract that can progress to malignancy [44–46]. HPV is the most common sexually transmitted viral infection and is well-known as the causative agent of cervical cancer. HPV infection has also been detected in the oral cavity. A large number of studies have demonstrated a 2–3-fold increase in the prevalence of HPV-driven oropharyngeal squamous cell carcinoma over the last three decades, particularly in North America and northern Europe [47–49]. Moreover, a recent review found that many studies have identified a high proportion of oral cancer with detectable HPV DNA [50]. Although the underlying reasons remain poorly understood, changes in sexual behavior, decreased rates of tonsillectomy performed in the pediatric population since the 70s, and progress in the diagnostic work-up and HPV testing assays have been proposed as the reasons [51–55]. Considering current trends, it is estimated that high-risk HPVs, such as HPV16 and HPV18, cause premalignant lesions [54, 55]. In the high-risk HPV types, E6 and E7 have been shown to function as oncoproteins [56, 57]. E6 and E7 oncoproteins are the key drivers of tumorigenesis by inactivating the tumor suppressors pRb and p53 [58].

Epstein–Barr virus (EBV) causes infectious mononucleosis and oral hairy leukoplakia, and is associated with various types of lymphoid and epithelial malignancies. During 1964, Epstein et al. determined the causative agent of Burkitt’s lymphoma to be a previously unknown member of the herpes family of viruses, later termed as EBV [59]. Burkitt lymphoma can be classified into three forms, which differ in geographic distribution and EBV association: endemic, sporadic, and HIV associated Burkitt lymphoma. Among them, endemic Burkitt lymphoma is associated with EBV in over 95 % cases [60]. Endemic Burkitt lymphoma is predominant in the equatorial belt of Africa and other parts of the world where malaria is hyperendemic [60–63]. On the other hand, although controversial, lymphoepithelial carcinoma and nasopharyngeal carcinoma may also be associated with EBV [64–68]. The association between lymphoepithelial carcinoma and EBV appears to differ according to geographic areas, race, and affected organs. In general, the association of lymphoepithelial carcinoma with EBV is strong in the head and neck region, whereas it is relatively weak at other sites. Moreover, the association is strong in East Asia and relatively weak in western countries. To a great extent, EBV-mediated disruption of cell growth checkpoints relies on direct modulation of cytokine receptor signaling mechanisms and alterations in the expression levels of various cytokines [69]. Moreover, EBV is associated with aggressive types of oral tumors, particularly in immunosuppressed patients [70]. Thus, EBV infection of the oral cavity is also associated with certain types of cancer. However, the pathogenesis of these tumors remains unclear.

Oropharyngeal candidiasis is an opportunistic infection primarily caused by *Candida albicans,* a ubiquitous fungal organism that is part of the normal microflora of the gastrointestinal and reproductive tracts. *Candida* species can be isolated as a commensal organism from the oral cavity in up to 80 % healthy individuals [71]. Depending on the host defense mechanisms or oral microenvironment, *Candida* species can transform from a harmless commensal species to pathogenic organisms causing oral mucosal infection [72, 73]. *Candida*-associated denture stomatitis affects more than 25 % denture wearers [74], and up to 90 % HIV^+^ patients have had at least one episode of oropharyngeal candidiasis [75]. When dealing with a hyperplastic epithelial lesion of the oral mucosa in which presence of *C. albicans* is demonstrated, it is referred to by many as *Candida*-associated leukoplakia or others prefer the term “hyperplastic candidiasis.” Chronic hyperplastic candidiasis showed a higher rate of malignant transformation on follow-up [76]. Animal studies have shown that *Candida* can cause epithelial hyperplasia and cellular atypia [77]. Strains of *Candida* can produce carcinogenic nitrosamine, *N*-nitrosobenzylmethylamine [78]. Thus certain strains of *Candida* play a key role in oncogene formation and initiation of cancer development [77–79]. However, the possible role of *Candida* in malignant transformation remains still unclear.

## Conclusion

Many of the causes of cancer are known to be closely linked to lifestyle factors, such as smoking, drinking, and diet. The oral environment is associated with the cause of various diseases, including cancer. The oral environment is influenced by various factors, including local and systemic factors. In the current review, in particular, we introduced the involvement of oral bacteria, funguses, and viruses in the development of cancer. During recent years, the role of oral care for the management of general health has been found to be important within the fields of medical and nursing care. Therefore, oral care for the management of the oral environment can be important for prevention of cancer.
